# Effect of 1.5% potassium oxalate on sensitivity control, color change, and quality of life after at-home tooth whitening: A randomized, placebo-controlled clinical trial

**DOI:** 10.1371/journal.pone.0277346

**Published:** 2022-11-17

**Authors:** Antonia Patricia Oliveira Barros, Danielle da Silva Pompeu, Elma Vieira Takeuchi, Cristiane de Melo Alencar, Eliane Bemerguy Alves, Cecy Martins Silva

**Affiliations:** 1 School of Dentistry, Federal University of Pará, Belém, Pará, Brazil; 2 Postgraduate Program in Dentistry of the Federal University of Pará, Belém, Pará, Brazil; 3 Araraquara School of Dentistry, Paulista State University (UNESP), Araraquara, São Paulo, Brazil; University of Catania: Universita degli Studi di Catania, ITALY

## Abstract

**Objective:**

This clinical trial evaluated the effect of 1.5% potassium oxalate (PO) in controlling sensitivity and color change after at-home tooth whitening. It also evaluated the influence of PO on health-related quality of life (HRQoL) and the degree of patient satisfaction after bleaching treatment.

**Materials and methods:**

Fifty volunteers were randomized into two groups (n = 25): At-home bleaching gel with 22% carbamide peroxide for 45 min + placebo gel (GP) or 1.5% PO (GPO) for 10 min. The intensity of tooth sensitivity was assessed daily through the visual analog scale. The color analysis was performed three times: baseline, 21 days, and 1 month after the last application of the whitening gel. The impact of the oral condition on the patient’s quality of life (OIDP) was used to measure the impact caused by the whitening treatment in relation to the individuals’ ability to carry out their daily activities and its influence on HRQOL.

**Results:**

No difference in tooth sensitivity was observed (p > 0.05). In addition, there was no difference in color change between groups (p > 0.05). However, there was an intragroup statistical difference throughout the evaluation period (p <0.05). The OIDP analysis showed a statistical difference between the groups (p > 0.05) and there was no difference between the groups regarding the degree of satisfaction with the bleaching (p > 0.05).

**Conclusions:**

The 1.5% PO was effective in preventing sensitivity and did not interfere with tooth whitening. Desensitizing therapy had a positive impact on quality of life and patient satisfaction.

## Introduction

Tooth whitening is considered a conservative treatment [1] and one of the most used procedures to treat dental discoloration, promoting satisfactory aesthetic results [2]. Carbamide peroxide (CP) is one of the active components used in whitening gels and is available in different concentrations [3, 4]. The oxidizing agent in CP is capable of diffusing through teeth enamel and dissociating into unstable free radicals (perhydroxyl, hydroxyl, and oxygen), which react with the macromolecules of organic pigments and triggering the breakdown of pigment carbon double bonds, making the tooth lighter [5].

A very common unwanted effect in at-home whitening treatment is sensitivity [6], which is usually reported in the first weeks of treatment [7] and is related to the number of free radicals from CP that reach the pulp through the dentinal tubules [8]. Some procedures have been used to reduce the pain caused by the whitening treatment, such as the use of whitening gels with a lower concentration and/or reduction of time and frequency of application [9] and the use of desensitizers [10–12].

Dentin desensitizers are classified according to their action, as neural and dentinal tubule blockers. Neural desensitizers act to stimulate nerve cells, interfering with the polarity of the cellular membrane and blocking the transmission of painful stimuli [13]. Dentinal tubule blockers occlude open dentinal tubules below the surface, interfering with dentinal fluid hydrodynamics, thus preventing dentin sensitivity [14]. Potassium oxalates are agents that have both actions: oxalate acts to form insoluble precipitates in the dentinal tubules, blocking the movement of dental fluid, and potassium acts to reduce the transmission of nerve impulses [15–17].

Among desensitizers, potassium oxalate has been widely used in clinical practice with satisfactory results [16, 18, 19]. Despite the possible benefit of associating the bleaching protocol with desensitization, it is not well-defined whether it could reduce the quality of bleaching if there is interference in the diffusion of peroxides [20].

To date, few clinical trials have investigated the influence of potassium oxalate after tooth whitening on health-related quality of life, psychosocial impact, and aesthetic self-perception [3]. Usually, studies published in the literature report the effectiveness of at-home tooth whitening, adverse effects, post-treatment sensitivity, and variables that result in varying concentrations of peroxide [21, 22]. The effect of this desensitizing treatment on patient-specific factors, such as perception, quality of life, and satisfaction, is an area that needs further investigation.

Therefore, the present study aimed to evaluate as a primary outcome the effect of 1.5% potassium oxalate in the control of pain sensitivity after at-home tooth whitening. In addition, the influence of potassium oxalate after bleaching treatment on color change, health-related quality of life (HRQoL), and patient satisfaction were analyzed as secondary outcomes. The tested null hypotheses are H01: There will be no difference in sensitivity between the whitened groups with CP at 22% associated or not with 1.5% potassium oxalate; H02: There will be no difference in color change between the groups whitened with CP at 22% associated or not with potassium oxalate at 1.5%, 30 days after the end of treatment; H03: Use of 1.5% potassium oxalate after whitening treatment will not influence HRQoL; H04: Use of potassium oxalate after whitening treatment will not influence patient satisfaction.

## Methods

### Ethical aspects

This clinical, randomized, double-blind, placebo-controlled study followed the guidelines of the “CONSORT” (Consolidated Standards of Reporting Trials) [23] and was approved by the Human Research Ethics Committee of the Health Sciences Institute of the Federal University of Pará under opinion 4.162.024. This study is available at Brazilian Clinical Trials Registry (http://www.ClinicalTrials.gov) under protocol NCT05028335. The participants were duly informed about the risks, methods, and objectives of this study. This term was prepared in accordance with the Declaration of Helsinki [24].

### Study design

This investigation was carried out through a clinical trial, randomized and double-blind, in which participants were unaware of the type of treatment they would undergo, just as the evaluator responsible for the statistical analysis was also unaware of the interventions carried out in the groups. Each research participant had their form coded so that there was confidentiality in the allocating process of the participants during the sample randomization for the different groups studied: (1) GP—application of a water-based placebo gel (KY^®^, Johnson & Johnson, São Paulo, SP, Brazil); (2) GPO—application of desensitizing gel (Potassium Oxalate 1.5%, BM4, Joinville, SC, Brazil). The daily assessment of pain sensitivity was self-reported by the research participants, during the 21-day follow-up of the study, using the visual analog scale (VAS). Dental color evaluation was measured using the Easyshade Advanced spectrophotometer (Vita-Zahnfabrik, Bad Säckingen, GE, Germany) at different evaluation periods. And a questionnaire based on the model of Masalu (2003) [25] and the modified model of Kothari et al. (2020) [26] was used to assess the health-related quality of life and the degree of satisfaction of the participants, respectively, before and after the completion of the bleaching treatment.

### Sample size

The sample size was calculated during a pilot study using the t test available in the G Power 3.1 software (Heinrich-Heine-Universität, Düsseldorf, Germany), with an effect size of 0.85 for testing difference in means. The calculation was performed considering 80% statistical power, a 2-sided significance level of 5%, and a 10% loss in sample size, resulting in 50 patients.

### Sample selection

Adult patients were recruited from January 12, 2021, to August 18, 2021. Participants included were examined and selected based on the following inclusion criteria: individuals aged 18 to 29 years old of both sexes with Shade higher than A2 in the maxillary incisors and canines, according to the Vita Classical color scale (Yorba Linda, CA, USA), absence of active caries lesions, patients who had never undergone whitening therapy, with good oral hygiene and with no hypersensitivity to tactile and evaporative stimuli through the Pain Visual Analog Scale. The following were excluded: patients undergoing fixed orthodontic treatment, patients with discolored non-vital teeth and/or with restorations in any elements, presence of cracks or fractures, patients allergic to the bleaching product, with gastroesophageal dysfunctions and with dentinal exposure in the anterior regions and/or teeth later.

Prophylaxis with a rubber cup and pumice stone was performed seven days before the start of the study in all participants. They received oral hygiene kits containing a toothbrush (Oral B, São Paulo, SP, Brazil) and toothpaste (My First Colgate^®^, Colgate-Palmolive Company, São Paulo, SP, Brazil) without desensitizing action and without fluoride, to avoid possible interference in the evaluations. Patients were instructed to use the kit daily, three times a day.

### Randomization

The randomization of the sample was performed by a researcher who did not participate in the clinical intervention stages of this study. Each volunteer had their medical records stored in coded envelopes to maintain allocation confidentiality during sample randomization. The randomization process was performed using the computer program BioEstat 5.0 (Civil Society, Mamirauá, Pará, Brazil), which used a computer-generated random table, 50 participants were allocated into two blocks (n = 25): a block with patients submitted to the use of placebo gel and another block with patients undergoing desensitizing therapy with 1.5% potassium oxalate. Thus, the following study groups were determined: GP and GPO (Table 1).

**Table 1:** Treatment used in different groups.

		Tooth bleaching	Placebo gel	Potassium Oxalate 1.5%
	**Manufacture/Composition**	Polanight, SDI (São Paulo, SP, Brazil) / 22% Carbamide Peroxide, Additives, Glycerol, Water, flavorings.	K-Y^®^, Johnson & Johnson (São Paulo, SP, Brazil)/ No active ingredient	Painless, BM4 (Maringá, PR, Brazil)/ Active ingredient: 1.5% Potassium Oxalate
**GROUPS**	**GP**	Use for 45 minutes from premolar to premolar in the tray	Application for 10 minutes	-
	**GPO**		-	Application for 10 minutes

### Blinding

In this double-blind study, volunteers were unaware of the desensitizing treatment used in each group. An evaluator, who did not know which group the collected data belonged to, performed the statistical analysis.

### Intervention

#### Homemade tooth whitening

Volunteers were recommended to apply one drop of the Polanight whitening agent (SDI, São Paulo, SP, Brazil) from premolar to premolar in the tray, which was used for 45 minutes, according to the manufacturer’s recommendations, within a period of 21 days by the study participants. The trays were made from plaster models, using an ethylene/vinyl acetate copolymer plate (FGM, Joinville, SC, Brazil) and a vacuum plasticizer (PlastVac P7/Bio Art, São Paulo, SP, Brazil).

#### Placebo

Participants in the placebo group (GP) were instructed to apply a drop of water-based placebo gel (K-Y^®^, Johnson & Johnson), present in a syringe identical to the desensitizing gel, without active ingredient, with a color, texture, and odor similar to 1.5% potassium oxalate gel, in the spaces related to the buccal surfaces of the teeth in the tray and use for 10 minutes during the 21 days of treatment, immediately after using the bleach. After removing the tray, the patients were instructed to brush their teeth with a fluoride-free toothpaste (My First Colgate^®^, São Paulo, SP, Brazil) and to clean the tray to remove all the gel inside.

#### Treatment with potassium oxalate at 1.5%

GPO participants were instructed to apply a small amount of 1.5% potassium oxalate gel (Painless, BM4, Joinville, SC, Brazil) in the spaces corresponding to the vestibular surfaces of the teeth, immediately after using the whitening gel, for 10 minutes and during the 21 days of treatment. After removing the tray, volunteers were instructed to brush their teeth with fluoride-free toothpaste (My First Colgate^®^, São Paulo, SP, Brazil) and clean the tray to remove all the gel from its interior.

### Outcomes primary

#### Sensitivity assessment

Sensitivity was assessed using a daily questionnaire provided to volunteers from the first session of at-home whitening treatment. Volunteers answered it during the 21 days of treatment, according to their perception of the pain threshold, the level of sensitivity or discomfort caused by the whitening treatment. The Visual Analog Scale (VAS) was adopted, ranging from 0 (no pain) to 10 (severe pain).

### Outcomes secondary

#### Color assessment

The color assessment was performed on the upper incisors and canines of each volunteer with the Easyshade Advanced spectrophotometer (Vita-Zahnfabrik, Bad Säckingen, GE, Germany), using the CIELAB system, where the color change values (ΔE) were obtained through the formula: ΔE = {(ΔL)^2^ + (Δa)^2^ + (Δb)^2^}^1/2^, where: ΔL* = L*- L*_0_; Δa* = a*-a*_0_; e Δb* = b*- b*_0_. To standardize the position of the device during color measurement, an additional silicone barrier (President Coltene, Rio de Janeiro, RJ, Brazil) was made with a circular cutout on the vestibular surface, with a diameter equivalent to the tip of the spectrophotometer. The color assessment was performed at three moments: before the whitening treatment, serving as a baseline; 21 days after treatment; and 1 month after the last application of the whitening gel.

#### Oral impact on daily performance (OIDP)

OIDP was used to measure the oral impact caused by the whitening treatment with the individuals’ ability to perform daily activities. It includes nine performances to be evaluated, based on the Masalu (2003) model [25], which analyzes physical, psychological, and social activities including eating, speaking, and pronouncing properly; cleaning teeth; sleeping and relaxing; smiling, laughing and showing your teeth without feeling ashamed and maintaining the emotional state without getting irritated. For each impact reported, the patient recorded the main symptom (1-tooth sensitivity, 2-tooth color, or 0-other reason). This questionnaire was provided before and after the whitening treatment.

#### Assessment of the patient’s degree of satisfaction with the whitening treatment

At the end of treatment, patients were asked to express themselves using a 7-point scale, based on the modified model by Kothari et al. (2020) [26], the degree of satisfaction with the treatment. The scale was ranked from 1 (not satisfied at all) to 7 (very satisfied). The participants also used the scale to express if they would recommend the whitening protocol to their family and friends, choosing from 1 (would not recommend) to 7 (highly recommend). The volunteers also answered a questionnaire with five questions regarding their satisfaction with the whitening protocol used and the results achieved with the treatment. For each question, the volunteers indicated one of the following scores: 1- completely agree; 2—partially agree; 3—no opinion; 4—somehow disagree, and 5—completely disagree.

### Data analysis

The data obtained in this study were tabulated in an Excel spreadsheet (Microsoft Windows 2010) and analyzed using the BioEstat^®^ software to verify the normality of the distribution using the Kolmogorov-Smirnov test. For the analysis of the primary outcome, the non-parametric Mann-Whitney test was used to assess sensitivity with comparison of mean sensitivity reported by patients using the Visual Analogue Scale, comparing the two groups (GP and GPO), once the data referring to sensitivity did not present a normal distribution. Secondary outcome analyses were performed using a two-way analysis of variance for repeated measures when analyzing the overall tooth color change (ΔE), as well as the values referring to the color dimensions (L*, a*, and b*) between the groups (GP and GPO), when the data showed a normal distribution; the Wilcoxon test to assess changes in health-related quality of life by the OIDP, and the Mann-Whitney test for independent samples to analyze data regarding the degree of satisfaction with color, as the data did not show a normal distribution. A significance level of 95% was considered for all analyses.

## Results

### Participants flow

**Fig 1:**
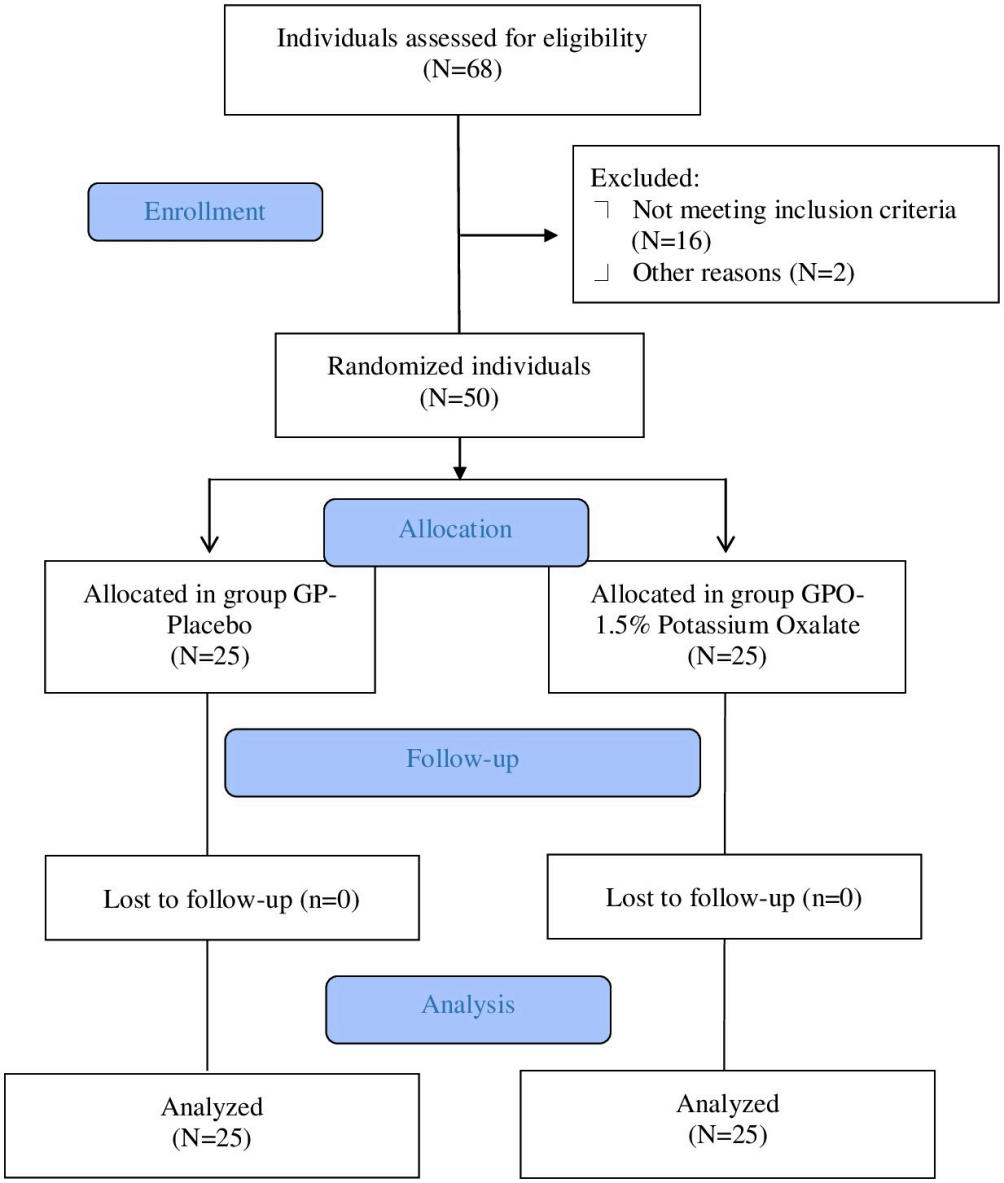
Study flow diagram.

Sixty-eight participants were evaluated, of which 50 were randomized, treated, and followed up (Fig 1).

In the final sample, there was a higher proportion of female volunteers (N = 27; 54%) when compared to male volunteers (N = 23; 46%). The mean age of the volunteers was 23 years (SD = 2.6; Range = 18–29). No significant difference was found in any of these characteristics between the different treatment groups (p > 0.05).

### Sensitivity assessment

**Fig 2:**
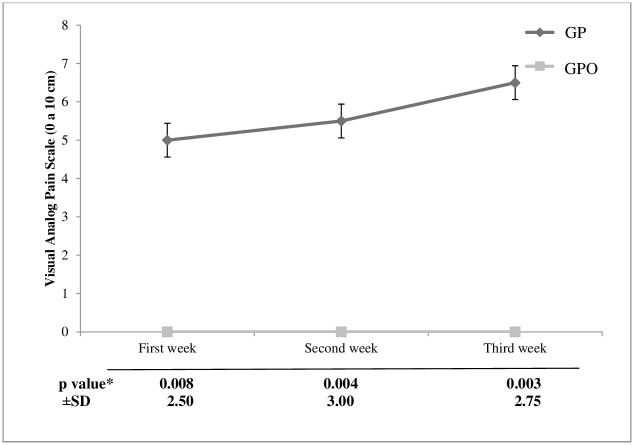
Means (standard deviation) of reported sensitivity level data using the visual analog scale. *Calculated by the Mann-Whitney test.

The placebo group (GP) resulted in a higher level of sensitivity during the whitening procedure. Volunteers from GP reported significantly higher sensitivity (p < 0.05) in all assessment periods when compared to GPO (Fig 2).

### Color assessment

**Fig 3:**
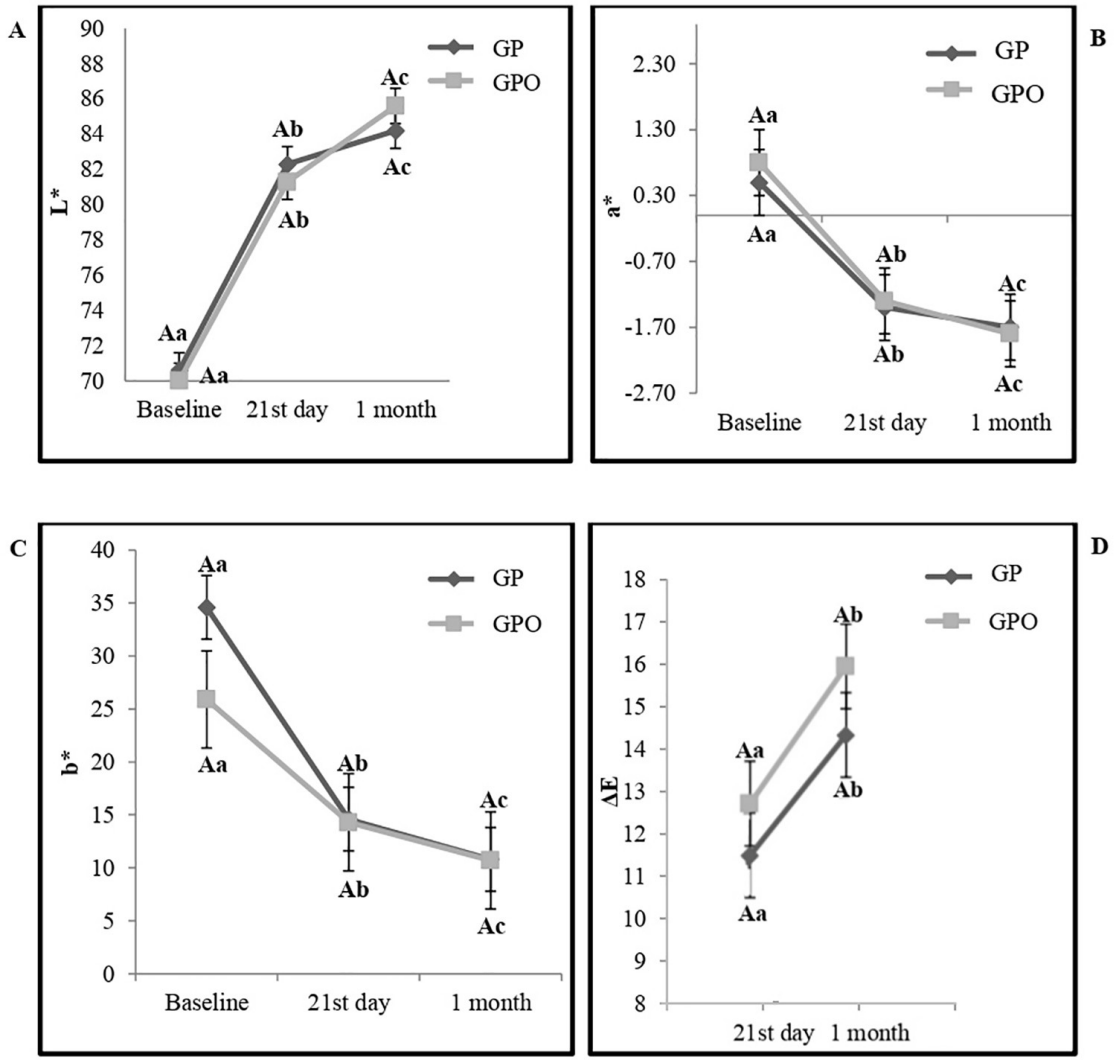
Color evaluation results for L* (A), a* (B), b* (C) and ΔE (D). * Different capital letters represent a statistically significant intergroup difference, according to the ANOVA Test (p≤ 0.05); *Different capital letters represent statistically significant intergroup difference (p≤0.05); **Different lowercase letters represent statistically significant intragroup difference (p≤0.05).

Color assessment results are shown in Fig 3. Intergroup comparisons between the variables “placebo and 1.5% oxalate” and “period of assessment” did not affect the values of L*, a*, b*, and ΔE. For intragroup comparisons, the assessment period affected L, a*, b*, and ΔE. During all assessments, tooth whitening resulted in lower values of a* and b*, with a significant difference in groups GP and GPO (p < 0.05). The lowest b* values were observed one month after the last session of home whitening treatment. In ΔE, ANOVA did not demonstrate any statistically significant difference in color change between the two groups in all evaluation periods. In both groups, there was a progressive increase in ΔE with a statistically significant difference between all assessment moments.

### Oral impact on daily performance (OIDP)

In the intragroup analysis evaluating sensitivity with patients’ quality of life (Table 2), GP showed a statistically significant difference (p<0.05) after bleaching treatment. However, GPO showed no significant difference. Intergroup analysis showed a significant difference between groups in the assessment of sensitivity in the OIDP after bleaching treatment.

**Table 2:** OIDP evaluating the sensitivity with the patients’ quality of life before and after the bleaching treatment.

GROUPS	OIDP Before Mean (±SD)	OIDP After	Difference in scores
*(N = 50)*			(After-Before)
**GP**	0.74 (±2.02)^**Aa**^	2.42 (±3.21)^**Ab**^	1.68
**GPO**	0.63 (±2.24)^**Aa**^	0.89 (±1.98)^**Ba**^	0.26

*Different capital letters represent a statistically significant intergroup difference, according to the Wilcoxon Test (p≤ 0.05);

*Different capital letters represent statistically significant intergroup difference (p≤0.05);

**Different lowercase letters represent statistically significant intragroup difference (p≤0.05).

The intragroup analysis evaluating the color of the teeth with the quality of life of the patients showed a significant difference (p < 0.05) before and after the bleaching treatment (Table 3). However, in the intergroup analysis, no significant differences were observed.

**Table 3:** OIDP evaluating the tooth color with the patients’ quality of life before and after the bleaching treatment.

GROUPS (N = 50)	OIDP Before Mean (±SD)	OIDP After	Difference in scores (After-Before)
**GP**	0.73 (±2.74)^**Aa**^	1.00 (±2.69)^**Ab**^	0.27
**GPO**	0.51 (±1.92)^**Aa**^	0.90 (±2.71)^**Ab**^	0.39

*Different capital letters represent a statistically significant intergroup difference, according to the Wilcoxon Test (p≤ 0.05);

*Different capital letters represent statistically significant intergroup difference (p≤0.05);

**Different lowercase letters represent statistically significant intragroup difference (p≤0.05).

### Patient satisfaction assessment

The assessment of patient satisfaction was obtained using a 7-point scale. Participants in both groups assigned similar scores (p = 0.99) when assessing their satisfaction after completing treatment at 21 days. Both groups were satisfied with the whitening treatment. High and similar scores between groups demonstrate that participants would certainly recommend a whitening treatment to their family and friends (p = 1.0). The answers to the questions evaluating the patients’ satisfaction with the whitening procedure performed are shown in Table 4. It was observed that there was a significant difference (p < 0.05) between groups GP and GPO only in the item referring to sensitivity resulting from whitening, with GPO in full agreement with this item. Both GP and GPO agreed that their teeth turned whiter than expected.

**Table 4:** Answers to questions assessing patient satisfaction with the bleaching procedure performed.

*Medians (1st/3rd quartiles) obtained for the following questions (scores 1 to 5)*	GP	GPO	
*Scores*: *1- totally agree*, *2- partially agree*, *3- no opinion*, *4- somehow disagree*, *5- totally disagree*			*P value* *
Were the procedures prior to placing the bleach comfortable?	1 (1.00/1.00)	1 (1.00/1.00)	1
Did you feel comfortable while bleaching?	1.5 (1.00/2.00)	1 (1.00/1.25)	0.99
Was the sensitivity of the bleaching less than expected?	4 (3.50/4.00)	1 (1.00/1.00)	0.028
Are the teeth lighter than you expected?	1 (1.00/1.25)	1 (1.00/1.00)	1
Am I satisfied with the final color of the teeth?	1 (1.00/1.25)	1 (1.00/1.00)	1

*Mann-Whitney test

## Discussion

Tooth sensitivity is a common adverse effect from a whitening treatment; [6] several desensitizing protocols [10–12] have been used to minimize it. Although potassium oxalate is used as a desensitizing alternative in tooth whitening [27], its influence on tooth color change is still unclear. In addition, the approach of this desensitization agent to the quality of life and patient satisfaction after bleaching treatment is still lacking in the literature.

The findings of the present study reported an increasing level of sensitivity during at-home bleaching treatment for the GP group. On the other hand, the GPO treated with a 1.5% potassium oxalate desensitizer did not report sensitivity throughout the treatment, rejecting the null hypothesis H01. The most accepted theory for sensitivity to bleaching is that of Markowitz [28], who argues that dentin sensitivity and sensitivity to bleaching have different mechanisms of pain generation, and sensitivity to bleaching is caused through direct activation by penetration of hydrogen peroxide in the intradental nerve [29].

According to Hodosh [30], there are desensitizers that act by blocking the tubules, which reduces the possibility of diffusion of whitening materials through the dentin, and those with neural action that prevent nerve stimulation, blocking the transmission of pain. Potassium oxalate has both neuronal and obliterating action [16]. The exact mechanism of action of potassium oxalate to reduce sensitivity in the whitening process is still unclear. It is known that oxalate initially acts as a carrier, allowing potassium contact and promoting depolarization of odontoblast endings, reducing dentin nerve activity [31] and consequently pain, which may explain the absence of pain reported by the GPO group. Godoy et al. (2021) [27] comparing 1.5% potassium oxalate with placebo in the control of sensitivity to tooth whitening obtained results equivalent to this study.

The results of this study showed that 1.5% potassium oxalate did not interfere with the color change in the GPO, accepting the null hypothesis H02. It is known that the clinical action of this desensitizing agent aims to reach the pulp-dentin complex, being especially applied on exposed dentin areas. However, when used after bleaching procedures, this product normally promotes coverage of enamel areas, therefore, doubts would arise as to the interference in the effectiveness of the bleaching procedure [32]. The groups demonstrated a color variation ΔE greater than or equal to 3.7 units in the CIELAB system. According to Costa et al. (2010) [33], this measure show an clinically perceptible color change in the different evaluation periods. This shows that potassium oxalate did not interfere with the bleaching efficacy of CP 22%. The literature reports that the size and area of the precipitated PO crystals depend on the concentration of the active agent, which can affect the occlusive power of the desensitizer [34]. Different concentrations of potassium oxalate gels ranging from 3% to 10% are described in the literature [35, 36]. In this study, 1.5% potassium oxalate was used, it may be that this concentration was not able to completely obliterate the dentinal tubules, and thus interfere with the effectiveness of the whitening product.

In this study, ΔE values varied satisfactorily during the 21 days of tooth whitening. Variations also occurred 1 month after the end of the whitening treatment, with an increase in ΔE, indicating that the whitening process continued throughout the days following treatment. Durán et al. (2018) [37] observed that this variation of ΔE tends to increase, due to the slow release of CP in the tooth and the stability of the tooth color. Tooth tissues, enamel, and dentin have distinct characteristics of opacity and translucency which are not monochromatic, but rather involve different colors in dispersion, requiring time to reach complete stability, justifying the increase in ΔE after completion of the whitening treatment in this study.

The L* coordinate represents the brightness from black (0) to white (100). In this study, there was an increase in this color parameter, indicating a variation of this coordinate to white. The translucent enamel allows light to go through the enamel prisms, with light reflection in dentin, which reflects more light when whitened, increasing the luminosity (value) [38] and explaining the increase in L*. For Meireles et al. (2008) [22], when looking at the three color coordinates separately, the L* values, which represent brightness, seem to be the most significant parameter. Torres et al. (2013) [39], reported that for tooth whitening to occur, an increase in the values in the L* chromatic axis must be observed, which consolidates the results obtained in this clinical trial.

The a* coordinate represents color and saturation on the red-green axis [38]. Positive values of a* indicate reddish tones and negative values indicate green tones [40]. The results showed a decrease in a* values, which means a shift to green. Pena et al. (2014) [41] reported that as the whitener acts on the tooth structure effectively, L*, a*, and b* color coordinates tend to change toward white, green, and blue, respectively. Vasconcelos et al. (2012) [42] stated that the change to green of the a* coordinate represents one of the indicators for tooth whitening, which agrees with the results of this clinical research.

The indicator of color and saturation on the blue-yellow axis is b*(with “b+” corresponding to yellow and “b-” to blue) [43]. In this study, a decline in b* was observed for the blue axis. Schmeling et al. (2012) [38] reported that the decrease in b* during whitening occurs because the process promotes the breakdown of the tooth’s chromatogenic molecules, which are responsible for the tooth’s yellowish tone. Thus, there is an increase in the bluish tone, removing the yellowish tone and bringing a lighter appearance to the tooth structure. These results corroborate previous studies [44, 45] that evaluated this coordinate as a parameter for color change in whitening.

The quality-of-life assessment aims to understand how external factors affect people’s daily activities [46]. When evaluating the subjective experiences of patients to determine the impact of oral health conditions, it was possible to observe the direct relationship of these factors with the treatment performed. When analyzing functional limitations: eating, relaxing, sleeping and how could be affected through the OIDP, it was observed that pain related to sensitivity resulting from the bleaching treatment negatively affected the functions of patients only in GP (group without desensitizing treatment after bleaching treatment). In addition, the results showed a positive impact on the quality of life of patients in both groups when evaluating the OIDP with tooth color before and after bleaching treatment, rejecting hypothesis H03.

The results of the OIDP questionnaire assessing sensitivity and color with quality of life before and after bleaching treatment in the analysis of functional limitations are quite surprising because it demonstrates a significant positive effect of desensitizing treatment on patients’ well-being. Patients with sensitivity report difficulties to relax and sleep [47], the short and sharp pain resulting from the adverse effects of tooth whitening generate discomfort with influence even when eating. Meireles et al. (2014) [48] reported that individuals dissatisfied with the color of their teeth feel embarrassed when smiling, negatively affecting their quality of life. Although there are studies in the literature that evaluated the impact of quality of life after home bleaching with CP on sensitivity and tooth color [49, 50], there are no findings that report the influence of OP on quality of life after bleaching treatment.

Patient satisfaction is a key factor to be analyzed, as it measures the efficiency of the whitening treatment from the patient’s point of view. In this study, both groups were satisfied with the treatment performed, rejecting hypothesis H04. Kothari et al. (2020) [26] reported that the initial result of whitening therapy is very important for patient satisfaction and cooperation, and that this satisfaction is directly related to the degree of whitening. A previous study [51] observed that tooth whitening with CP promoted a satisfaction of 92% to 100% of patients. It is known that the color of teeth, in general, is a physical phenomenon that depends on three factors: light source, object and observer, the latter being responsible for the subjectivity of color and for the emotions involved [52]. Color causes different sensations and emotions for each observer, which results in the satisfaction or lack of it that each person presents with the aesthetics of their teeth. It is noteworthy that there are no findings in the literature evaluating the degree of patient satisfaction when using 1.5% potassium oxalate gel associated with homemade whitening treatment with CP.

Monitoring the patient over time is necessary to analyze the efficacy of 1.5% potassium oxalate gel, which is a limitation of this study. We suggest the development of future long-term studies in other populations to explain the hypothetical group differences.

## Conclusion

The 1.5% potassium oxalate gel prevented sensitivity, did not interfere with the effectiveness of at-home whitening with 22% CP, and had a positive impact on the quality of life and the degree of patient satisfaction.

## Supporting information

S1 Table 2010-CONSORT checklist. (DOCX)

S1 Protocol Copy of the protocol of the complete and detailed study project approved by the ethics committee original language. (DOC)

S2 Protocol Copy of the protocol of the complete and detailed study project approved by the ethics committee translated into English. (DOC)
