# Supplementary material for: Effect of 1.5% potassium oxalate on sensitivity control, color change, and quality of life after at-home tooth whitening: A randomized, placebo-controlled clinical trial
Source: PLoS One. 2022 Nov 17;17(11):e0277346. doi: 10.1371/journal.pone.0277346 (PMC9671445; doi:10.1371/journal.pone.0277346)
Supplement: S1 Protocol — (DOC) [file pone.0277346.s002.doc]

**PROJETO DE PESQUISA**

**1 - IDENTIFICAÇÃO DO PROJETO**

TÍTULO DO PROJETO: Efeito do uso do oxalato de potássio a 1,5% no controle da sensibilidade dentária e alteração de cor pós-clareamento caseiro: estudo clínico, randomizado, controlado por placebo.

GRANDE ÁREA DE CONHECIMENTO: (Ciências da Saúde)

ÁREA DE CONHECIMENTO: (Odontologia (4.02.00.00.0)

SUBÁREA: Dentística (4.02.04.000)

INSTITUIÇÃO: Universidade Federal do Pará

CENTRO / DEPARTAMENTO: Centro de ciências da saúde/Faculdade de Odontologia

UNIDADE EXECUTORA: Faculdade de odontologia

ENDEREÇO: Av. Augusto Corrêa, nº 01 – Cidade Universitária José da Silveira Netto

| MUNICÍPIO  Belém | CEP  66640480 | U.F.  PA | TEL/FAX  91-32017494 | E-MAIL  [cecymsilva@gmail.com](mailto:cecymsilva@gmail.com) |
| --- | --- | --- | --- | --- |

COORDENADOR DO PROJETO: CECY MARTINS SILVA

DEPARTAMENTO: FACULDADE DE ODONTOLOGIA

OUTRAS INSTITUIÇÕES PARTICIPANTES

**2 – EQUIPE DO PROJETO**

| **Matrícula** | **Nome completo** | **Tipo*** | **Titulação máxima** | **Departamento** | **Função no projeto**** | **Carga horária no projeto** |
| --- | --- | --- | --- | --- | --- | --- |
| 0327584 | Cecy Martins Silva | PE | Doutor | Faculdade de odontologia | CD | 5h |
| 1259040 | Eliane Bemerguy Alves | PE | Doutor | Faculdade de odontologia | CL | 5h |
|  | Antonia Patricia Oliveira Barros |  | Estudante de Iniciação científica | Faculdade de odontologia | CL | - |
|  | Danielle da Silva Pompeu |  | Estudante de Iniciação científica | Faculdade de odontologia | CL | - |
|  | Elma Vieira Takeuchi |  | Estudante de mestrado | Faculdade de odontologia | CL | - |
|  | Cristiane de Melo Alencar |  | Estudante de doutorado | Faculdade de odontologia | CL | - |

* TA: Técnico Administrativo ** CD: Coordenador

PV: Professor Visitante CL: Colaborador

PE: Professor Permanente (lotado no centro em que pertence o projeto) CS: Consultor

PP: Professor Participante (lotado em outro centro)

PPE: Professor Participante Externo

TE: Técnico Administrativo Externo

PB: Professor Bolsista de Agência de Fomento (CAPES, CNPQ, DAAD, etc..)

**ROJETO DE PESQUISA**

**3 - INTRODUÇÃO**

O clareamento dental é considerado um tratamento conservador para remoção de pigmentos que promovem o escurecimento indesejado dos dentes, ^1,2,3^ e é preconizado para tratar pacientes que buscam melhorias na aparência dental, pois, apresenta uma abordagem minimamente invasiva quando comparada a outros tratamentos estéticos invasivos. ^4^ O peróxido de carbamida (PC) é um dos componentes ativos empregados no gel clareador, o qual é disponibilizado em diferentes concentrações.^1,5^ O agente oxidante presente no PC é capaz de se difundir pelo esmalte dental e se dissociar nos radicais livres instáveis (peridroxil, hidroxila e oxigênio) os quais reagem com as macromoléculas dos pigmentos orgânicos desencadeando a quebra das ligações duplas de carbono dos pigmentos, resultando em moléculas menores que proporcionam uma mudança no espectro de absorção, deixando o dente mais claro. ^5^

Um efeito indesejado bastante comum durante o tratamento clareador caseiro, é a sensibilidade dental.^6,7,8,9^ Essa sensibilidade é relatada por ocorrer, geralmente, nas primeiras semanas do tratamento.^10,11^ Esta sensibilidade, está relacionada à quantidade de radicais livres do peróxido de carbamida que chegam à polpa através dos túbulos dentinários.^12,2,13,14^ Para redução da dor causada pelo tratamento clareador, alguns métodos podem ser utilizados, como o uso de géis com menor concentração e/ou redução do tempo, frequência de aplicação do gel clareador,^12,2^ administração de analgésicos/antiinflamatórios^15^ e a utilização de dessensibilizantes.^5,9,16,3^

Muitos agentes com diferentes mecanismos de ação foram descritos e avaliados na literatura relacionada ao manejo da DH. Os dessensibilizadores de dentina podem ser classificados, de acordo com sua ação, como os neurais (por exemplo, nitrato de potássio e lasers de baixa potência), que atuam na estimulação das células nervosas, mais especificamente a bomba Na + / K + na membrana celular, interferindo na polaridade da membrana celular, aumentando a amplitude do potencial de ação da membrana, e bloqueando a transmissão de estímulos dolorosos, ^17^ os bloqueadores de túbulos dentinários, por exemplo, oxalatos, glutaraldeído e lasers de alta potência, na qual geram precipitados capazes de ocluir os túbulos dentinários abertos abaixo da superfície, interferindo na hidrodinâmica do fluido dentinário, evitando, assim, a sensibilidade da dentina,^18^ e os agentes com ambas as ações (por exemplo, o oxalato de potássio), na qual o oxalato tem a ação de formar precipitados insolúveis nos túbulos dentinários bloqueando a movimentação do fluido dentário, e o potássio que age na redução da transmissão de impulsos nervosos.^19^

Entre os dessensibilizadores, o oxalato de potássio tem sido amplamente utilizado na prática clínica, apresentando resultados satisfatórios, sem efeitos colaterais.^20,21,22^ O mecanismo de ação desse agente é baseado na obliteração dos túbulos de dentina expostos, devido a precipitação de cristais de oxalato de potássio ^23^ e à despolarização das terminações nervosas.^17^ Em uma revisão sistemática da literatura, na qual foi avaliado a eficácia de diferentes agentes dessensibilizantes na redução da sensibilidade dentinária, foi observado, com base nos resultados dos autores, ^24^ que o oxalato de potássio obteve efeito significativo na redução da dor. Até o presente momento, não existem relatos na literatura de estudos clínicos que avaliaram o efeito do oxalato de potássio a 1,5% no controle da sensibilidade pós-clareamento caseiro com peróxido de carbamida a 22%, justificando os objetivos desta investigação científica.

A cor do dente é considerada um fenômeno de grande complexidade influenciada por fatores como as condições de iluminação ambiente, translucidez, opacidade, espalhamento da luz, brilho e ainda pelas estruturas e processos morfofisiológicos da visão que variam em cada indivíduo.^25^ A percepção da cor é subjetiva e necessita de um apoio mais quantitativo para ser avaliada. O espectrofotômetro é um equipamento capaz de medir o comprimento de onda de uma determinada radiação a partir da refletância ou transmitância de um objeto.^26^ Neste estudo, iremos utilizar o espectrofotômetro EasyshadeAdvanced(Vita-Zahnfabrik, Alemanha), utilizado o sistema CIE L*a*b*, para avaliar alteração de cor entre os grupos clareados com peróxido de carbamida a 22%. Vale ressalta que, o sistema de cor CIEL*a*b* é capaz de converter a resposta do olho humano às cores, de forma que ela seja eletronicamente mensurada. ^26^

**4 – JUSTIFICATIVA**

Os oxalatos, em particular, têm uma longa história no tratamento da DH ^27,28,29^ e são amplamente reconhecidos como capazes de reduzir a sensibilidade pós-operatória.^30^ O tratamento com produtos à base de oxalato é capaz de diminuir a permeabilidade da dentina,^31,32,33^ formar precipitados nos túbulos da dentina,^34^  bloquear o fluxo de fluidos ^35,36^ e reduzir a dor relacionada à DH.

Os precipitados de oxalato também são relativamente insolúveis em ácido, tornando-os resistentes à dissolução no ambiente oral.^37^ A combinação de potássio e oxalatos foi introduzida para DH no final da década de 1970^31^ e produziu dois efeitos principais: um efeito inicial, a partir dos íons potássio, caracterizado por despolarização neutra; e efeito tardio, resultante da obstrução dos túbulos pela formação de cristais de oxalato de cálcio.^38^ Este último é baseado na troca de íons entre os íons metálicos presentes no oxalato (potássio) e os (Ca2 +) do fluido dentinário. A troca de Ca2 + por K + leva à formação de uma camada cristalina de oxalato de cálcio 'levemente solúvel', que é depositada na superfície da dentina.

O sistema de cor – CIE L*a*b*, na qual será utilizado neste estudo, toma por base a teoria da percepção das cores dos três receptores nos olhos (três tipos de cones da retina) separados pelas cores primárias– vermelho, verde, azul.^26^ Através desse sistema o espectro de refletância é filtrado de forma que o espectro do matiz vermelho, verde e azul é extraído como três coordenadas, e processadas para dar a resposta L*a*b*.^39^ As letras da sigla CIE L*a*b* relacionam-se às três direções na esfera de cor : L* significa luminosidade da cor, as coordenadas cromáticas a*(representam vermelho– verde) e b* (representam amarelo- azul), são perpendiculares ao L * e partem em quatro direções ortogonais (a+): caracterizando a cor avermelhada; (a-): caracterizando a cor verde; (b+): caracterizando uma tendência ao amarelo; e (b-): com uma tendência de cor ao matiz do azul.^26,27^

Além dos atributos a* e b* a combinação deles também determina parâmetros de Croma (C*) e H* (Matiz).^27^ O croma é a saturação da cor mensura a distância do eixo L* no referido sistema e representa a mudança de um estado opaco, mais apagado para uma cor saturada, viva. O Matiz (H *) é uma medida de ângulo que determina o comprimento de onda predominante que compõe aquela cor (matiz predominante). ^39^ A cor pode ser determinada pela combinação de efeitos de coloração intrínseca e extrínseca, a primeira relacionada ao espalhamento da luz e às propriedades de absorção do esmalte e da dentina, determinadas por suas condições morfofisiológicas e a outra associada a absorção de pigmentos na superfície do esmalte dental. ^40^

Este estudo clínico, randomizado, controlado por placebo, portanto, contribuirá positivamente para a elucidação da efetividade do oxalato de potássio a 1,5% no controle da sensibilidade dolorosa, após tratamento clareador caseiro, bem como na percepção da alteração de cor entre os grupos clareados com peróxido de carbamida a 22%.

**PROJETO DE PESQUISA**

**5 – OBJETIVOS**

Este ensaio clínico, randomizado, controlado por placebo visa avaliar o efeito do oxalato de potássio à 1,5% no controle da sensibilidade dolorosa, e na alteração de cor após clareamento dental caseiro. As hipóteses nulas testada no presente estudo serão: H01 - Não haverá diferença na sensibilidade dentária entre os grupos clareados com peróxido de carbamida à 22% associados ou não ao oxalato de potássio à 1,5%, trinta dias após o final dos tratamentos.

H02- Não haverá diferença na alteração de cor entre os grupos clareados com peróxido de carbamida à 22% associados ou não ao oxalato de potássio à 15%, trinta dias após o final dos tratamentos.

H03- Uso de oxalato de potássio a 1,5% após tratamento clareador não influenciará na Qualidade de Vida Relacionado a Saúde (QVRS).

H04- Uso de oxalato de potássio após o tratamento clareador não influenciará na satisfação do paciente.

**6 – METODOLOGIA**

## **6.1. Aspectos éticos**

Este projeto de pesquisa seguirá as recomendações do *“consort”* (consolidated standards of reporting trials). Os voluntários da pesquisa serão devidamente esclarecidos e informados sobre os riscos, métodos e objetivos deste projeto, sendo necessária a assinatura do termo de consentimento livre e esclarecido – tcle (anexo I), em conformidade com a declaração de Helsinki.^41^

Todas as informações a serem colhidas terão finalidade exclusivamente científica e a identidade dos voluntários será preservada. A participação no estudo poderá ser cancelada e o termo de consentimento retirado a qualquer momento da pesquisa, sendo assegurado o sigilo de confidencialidade do voluntário mesmo em caso de abandono do tratamento.

## **6.2. Seleção da amostra**

Serão selecionados cinquenta voluntários com idade entre 18 e 29 anos que deverão seguir os critérios de inclusão e exclusão descritos na tabela 1. A avaliação clínica dos voluntários será realizada através da anamnese, exame clínico intraoral e registro da cor. Todos os participantes serão submetidos à profilaxia realizada com taça de borracha (Microdont, SP, Brasil) e pedra pomes (Biodinâmica, PR, Brasil) sete dias antes do início do estudo e receberão kits de higiene bucal, para uso durante todo o tratamento, para padronização de um dentifríco que não possua ação dessensibilizante e que não contenha fluór, a fim de amenizar possíveis interferências na avaliação deste estudo. O kit será composto de uma escova dental (Oral B, Cerdas Indicator, São Paulo, SP, Brasil) e um dentifrício (My First Colgate®, Colgate-Palmolive Company, SP, Brasil), sob orientação para uso três vezes ao dia.

**Tabela 1:** Críterios de inclusão e exclusão.

| ***CRITÉRIOS DE INCLUSÃO*** | ***CRITÉRIOS DE EXCLUSÃO*** |
| --- | --- |
| - Tonalidade maior que A2 nos incisivos e caninos superiores, de acordo com a escala de cores Vita Classical; - Ausência de lesões de cárie ativa; - Pacientes que nunca tenham sido submetidos à terapia clareadora - Apresentar uma boa higiene oral; - Não apresentar hipersensibilidade aos estímulos tátéis e evaporativos através da Escala Visual Analógica de dor; - Não ser fumante; - Não estar grávida; | - Pacientes em tratamento ortodôntico fixo; - Dentes não vitais com escurecimento; - Presença de trincas ou fraturas; - Pacientes alérgicos ao produto; - Restaurações extensas em molares; - Presença de disfunções gastroesofágicas; - Presença de exposição dentinária em dentes anteriores e/ou posteriores; - Pacientes acima de 30 anos de idade. |

## **6.3. Desenho de estudo**

Este estudo clínico será randomizado, controlado por placebo e cego. Para a determinação dos grupos, será realizado um processo de randomização que determinará o tratamento a ser aplicado. Os pacientes serão randomizados e distribuídos aleatoriamente em dois grupos: G1- Placebo; G2 – Oxalato de Potássio; como descrito na figura 1.

Pacientes Avaliados (N=X)

Pacientes Excluídos Y=(X-50)

**Recrutamento**

Randomização por sorteio (N=50)

Tratamento clareador

Peróxido de Carbamida 22% Polanight (SDI)

**Acompanhamento**

**G1 (n=25)**

Placebo

(dentifrício sem flúor)

**G2 (n=25)**

Grupo Experimental

(Oxalato de Potássio a 1,5%)

Avaliação da dor pelo questionário diário

(n=50)

**Análises**

**Figura 1**: Desenho do estudo

Todos os grupos serão submetidos ao tratamento clareador caseiro com peróxido de carbamida 22% Polanight (SDI). Somente o grupo G2 receberá tratamento dessensibilizante com gel oxalato de potassio a 1,5% (tabela 2). Os voluntários serão orientados quanto ao uso do tratamento clareador e tratamento com placebo ou dessensibilizante.

**Tabela 2.** Divisão dos grupos, tratamento clareador, tratamentos dessensibilizante.

| ***GRUPOS (N=50)*** | ***TRATAMENTO CLAREADOR*** | ***TRATAMENTO DESSENSIBILIZANTE*** |
| --- | --- | --- |
| **G1** |  | ---- |
| **G2** | Pola Night  22% PC  (SDI) | Oxalato de Potássio a 1,5% (Painless, BM4,SC, Brasil) |

## **6.4. Randomização**

O processo de randomização será realizado mediante sorteio numérico pelo pesquisador principal. Será atribuído um número para cada grupo experimental (1 para G1, 2 para G2) e para cada voluntário será executado um sorteio. Os pacientes serão numerados de acordo com a sequência de inscrição.

## **6.5. Cegamento**

Neste estudo duplo-cego, o participante desconhecerá o tratamento dessensibilizante que irá receber, pois, tanto o gel dessensibilizante quanto o gel placebo serão inseridos em recipiente iguais para impossibilitar a sua identificação. A textura, cor e odor do placebo será similar ao oxalato de potássio a 1,5% (Painless, BM4,SC, Brasil). O avaliador da sensibilidade dolorosa também desconhecerá o grupo ao qual o participante pertencerá, porque não participará do processo de randomização. A pesquisa contará com um único operador, que executará a parte experimental.

**6.6 Intervenção**

Cada voluntário do grupo receberá um kit contendo uma bisnaga com agente clareador Polanight (SDI, SP, Brasil), duas moldeiras individuais de copolímero etileno/ acetato de vinila (superior e inferior), um tubo sem identificação com o dessensibilizante (oxalato de potássio à 1,5%), uma escova dental e um dentifrício sem flúor (My First Colgate®, Colgate-Palmolive Company, SP, Brasil).

## **Clareamento dentário**

Os voluntários serão moldados com alginato (Jeltrate Plus - Dentsply) com o auxílio de uma moldeira (Tecnodent). Em seguida, os modelos serão confeccionados empregando gesso especial (Durone- Dentsply). As moldeiras serão confeccionadas a partir dos modelos obtidos utilizando uma placa de copolímero etileno/ acetato de vinila (FGM) e uma plastificadora a vácuo (Plastivac P7/Bio Art).

Será recomendada a aplicação de uma gota do agente clareador Polanight (SDI, SP, Brasil) em cada dente correspondente na moldeira que deverá ser utilizada durante 45 minutos por dia no período de vinte e um dias.

## -*Placebo*

Após o tratamento clareador, as moldeiras serão lavadas com água corrente, e os voluntários do grupo 1 aplicarão uma pequena quantidade do gel placebo, sem princípio ativo, com cor, textura e odor semelhante ao gel de oxalato a 1,5% (Painless, BM4, SC, Brasil) nos espaços relativos às porções vestibulares dos dentes das moldeiras, que deverão ser utilizadas por 10 minutos. Após a remoção da moldeira, o paciente deverá escovar os dentes com o dentifrício sem flúor (My First Colgate®, Colgate-Palmolive Company, SP, Brasil).

*- Tratamento com Oxalato de Potássio à 1,5%*

Após o tratamento clareador, as moldeiras serão lavadas com água corrente, e os voluntários do grupo 1 aplicarão uma pequena quantidade do gel de oxalato a 1,5% (Painless, BM4, SC, Brasil) nos espaços relativos às porções vestibulares dos dentes das moldeiras, que deverão ser utilizadas por 10 minutos. Após a remoção da moldeira, o paciente deverá escovar os dentes com o dentifrício sem flúor (My First Colgate®) e realizar a higienização da moldeira, a fim de remover todo o gel do interior da moldeira.

## **6.9. Avaliação da Cor**

A avaliação de cor será realizada nos incisivos e caninos superiores de cada voluntário com um espectrofotômetro EasyshadeAdvanced(Vita-Zahnfabrik, Alemanha), utilizado o sistema CIE L*a*b*, onde os valores da alteração de cor (ΔE) foram obtidos para cada grupo de dentes por meio da fórmula: ΔE = {(ΔL)^2^ + (Δa)^2^ + (Δb)^2^}^1/2^ , onde: ΔL* = L*- L*_0_; Δa* = a*-a*_0_; e Δb* = b*- b*_0_. A avaliação de cor será realizada em três tempos: antes do tratamento clareador servindo de linha de base, 21 dias e 1 mês após a última aplicação do gel clareador.

### **6.10. Avaliação da sensibilidade**

A sensibilidade pós-operatória será avaliada por meio de um questionário diário que será fornecido aos voluntários a partir da primeira sessão de tratamento clareador caseiro, o qual, deverá ser preenchido durante os 21 dias de tratamento, segundo sua percepção pessoal e limiar a dor, sobre o nível de sensibilidade ou desconforto causado pelo tratamento clareador, em uma escala de 0 (ausencia de dor) a 10 (dor severa), de acordo com a Escava Visual Analógica.

**6.11. Avaliação do Impacto oral no desempenho diário (OIDP)**

O OIDP sera usado para medir o impacto bucal causado pelo tratamento clareador na capacidade dos indivíduos de realizar as atividades diárias. Inclui nove performances a serem avaliadas, com base no modelo de Masalu (2003),^42^ que analisa atividades físicas, psicológicas e sociais, incluindo comer, falar e pronunciar corretamente; limpeza dos dentes; dormir e relaxar; sorrindo, rindo e mostrando os dentes sem sentir vergonha e mantendo o estado emocional sem se irritar. Para cada impacto relatado, o paciente irá registrar o sintoma principal (1- sensibilidade de dente, 2- cor do dente ou 0- outro motivo). Este questionário será fornecido antes e após o tratamento clareador.

**6.12. Avaliação** **do grau de satisfação do paciente com o tratamento clareador**

Ao final do tratamento, os pacientes serão solicitados a se expressarem por meio de uma escala de 7 pontos, baseada no modelo modificado de Kothari et al. (2020),^43^ o grau de satisfação com o tratamento. A escala será classificada de 1 (nada satisfeito) a 7 (muito satisfeito). Os participantes também utilizarão a escala para expressar se recomendariam o protocolo de clareamento para seus familiares e amigos, escolhendo de 1 (não recomendaria) a 7 (recomendaria). Os voluntários também responderão a um questionário com cinco perguntas sobre sua satisfação com o protocolo de clareamento utilizado e os resultados alcançados com o tratamento. Para cada questão, os voluntários indicarão uma das seguintes pontuações: 1- concordo totalmente; 2 - concordo parcialmente; 3 - sem opinião; 4 - discordo de alguma forma e 5 - discordo totalmente.

## **6.13. Análise Estatística**

Os valores da sensibilidade referida pelos voluntários serão tabulados em uma planilha Excel (Microsoft Windows 2010) e analisados utilizando o programa BioEstat.®. Caso este estudo gere dados não paramétricos, será realizado o teste de Friedman para análise intragrupo e teste de Wilcoxon para análise intergrupo. Para todas as análises serão considerados os níveis de significância de 5%.

**PROJETO DE PESQUISA**

**7 – METAS**

- O levantamento bibliográfico ocorrerá de agosto de 2020 a dezembro de 2021;

- Após a aprovação no Comitê de Bioética, será iniciado ensaio clínico randomizado: o recrutamento, randomização e alocação dos voluntários, realização dos tratamentos, avaliação da sensibilidade e alteração de cor ocorrerão no período de agosto de 2020 à setembro de 2021.

- Tabulação e análise estatística dos resultados será realizada no período entre outubro à dezembro 2021;

- A Elaboração e entrega do relatório final ocorrerá até dezembro de 2021

**8 - BIBLIOGRAFIA**

1. Abouassi T, Wolkewitz M, Hahn P. Effect of carbamide peroxide and hydrogen peroxide on enamel surface: an in vitro study. Clin Oral Invest. 2011; 15:673–680.

2. Basting RT, Amaral FLB, França FMG, Flório FM. Clinical comparative study of the effectiveness of and tooth sensitivity to 10% and 20% carbide peroxide home-use and 35% and 38% hydrogen peroxide in-office bleaching materials containing desensitizing agents. Oper Dent. 2012; 37(5):464- 473.

3. Tay LY, Kose C, Loguercio AD, Reis A. Assessing the effect of a desensitizing agent used before in-office tooth bleaching. JADA. 2009; 140(10): 1245-1251.

4 Alani A, Kelleher M, Hemmings K, Saunders M, Hunter M,Barclay S, Ashley M, Djemal S, Bishop K, Darbar U (2015)Balancing the risks and benefits associated with cosmetic dentistry–a joint statement by UK specialist dental societies. Br Dent J218(9):543–548. https://doi.org/10.1038/sj.bdj.2015.345.

5Alqahtani MQ. Tooth-bleaching procedures and their controversial effects: A literature review. Saudi Dent J. 2014; 26:33-46.

6. Bonaf E, Bacovis LB, Iensen S, Loguercio AD, Reis A, Kossatz S. Tooth sensitivity and efficacy of in-office bleaching in restored teeth. J Dent. 2013; 41:363-369 .

7. Haywood VB, Leech T, Heymann HO, Crumpler D, Bruggers K. Nightguard vital bleaching: effects on enamel surface texture and diffusion. Oper Dent. 1990; 21(10): 801-804.

8. Kossatz S, Dalanhol AP, Cunha T, Loguercio A, Reis A. Effect of light activation on tooth sensitivity after in-office bleaching. Oper Dent. 2011; 36(3): 251-257.

9. Reis A, Dalanhol AP, Cunha TS, Kossatz S, Loguercio AD. Assessment of tooth sensitivity using a desensitizer before light-activated bleaching. Oper Dent. 2011; 36(1): 12-17.

10. Browning WD, Cho SD, Deschepper EJ. Effect of a nano- hydroxyapatite paste on bleaching related tooth sensitivity. J Esthet Restor Dent. 2012; 24(4):268-276.

11. Po LH & Wilson N. Effects of differents desensitizing agents on bleaching treatments. EJGD. 2014; 3(2): 93-99.

12. Armênio RV, Fitarelli F, Armênio MF, Demarco FF, Reis A, Loguercio AD. The effect of fluoride gel use on bleaching sensitivity: a doubleblind randomized controlled clinical trial. JADA. 2008; 139(5):592-597.

13. Camargo SEA, Valera MC, Camargo CHR, Mancini MNG, Menezes MM. Penetration of 38% hydrogen peroxide into the pulp chamber in bovine and human teeth submitted to office bleach technique. JOE. 2007; 33(9):1074-1077.

14. Costa CAS, Riehl H, Kina JF, Sacono NT, Hebling J. Human pulp response to in-office tooth bleaching. Oral Surg Oral Med Oral Pathol Oral Radiol Endod. 2010; 109:59-64.

15. Charakorn P, Cabanilla LL, Wagner WC, Foong WC, Shaheen J, Pregitzer R, Schneider D. The effect of preoperative ibuprofen on tooth sensitivity caused by in-office bleaching. Oper Dent. 2009; 34:131–135.

16. Cerqueira RR, Hofstaetter FL, Rezende M, Martins GC, Loguercio AD, Reis A, Kossatz S. Efeito do uso de agente dessensibilizante na efetividade do clareamento e na sensibilidade dental. Rev Assoc Paul Cir Dent. 2013; 67(1):64-67.

17- Wakabayashi H, Hamba M, Matsumoto K, Tachibana H (1993) Effect of irradiation by semiconductor laser on responses evoked in trigeminal caudal neurons by tooth pulp stimulation. Lasers Surg Med 13:605–610

18. A. Davari, E. Ataei, H. Assarzadeh, Dentin hypersensitivity: etiology, diagnosis and treatment; a literature review, J. Dent. 14 (3) (2013) 136–145.

19. E. Oncu, S. Karabekiroglu, N. Unlu, Effects of different desensitizers and lasers on dentine tubules: an in-vitro analysis, Microsc. Res. Tech. 80 (7) (2017) 737–744

20. D.G. Gillam, H.S. Seo, H.N. Newman, J.S. Bulman, Comparison of dentine hypersensitivity in selected occidental and oriental populations, J. Oral Rehabil. 28 (1) (2001) 20–25.

21. J. Pereira, A. Martineli, S. Santiago, Treating hypersensitive dentin with three different potassium oxalate-based gel formulations: a clinical study, J. Appl. Oral Sci. 9 (2001) 123–130.

22. S. Sauro, M.G. Gandolfi, C. Prati, R. Mongiorgi, Oxalate-containing phytocomplexes as dentine desensitisers: an in vitro study, Arch. Oral Biol. 51 (8) (2006) 655–664.

23. E.M. Varoni, T. Zuccheri, A. Carletta, B. Palazzo, A. Cochis, M. Colonna, L. Rimondini, In vitro efficacy of a novel potassium oxalate hydrogel for dentin hypersensitivity, Eur. J. Oral Sci. 125 (2) (2017) 151–159.

24- Ayad F, Ayad N, Yun Po Zhang YP, De Vizio W, Cummis D, Mateo LR. Comparing the efficacy in reducing dentin hypersensitivity of a new toothpaste containing 8.0% Arginine Calcium Carbonate, and 1450 ppm fluoride to a commercial sensitive toothpaste containing 2% potassium ion: an eight-week clinical study on Canadian adults. J Clin Dent. 2009;20(1):10-6.

25 -JOINER,A. Tooth color : a review of the literature. Journal of Dentistry,v.32, p.3-12, 2004.

26. P. Amini, M. Miner, P.A. Sagel, R.W. Gerlach, Effects of 1.5% oxalate stripes versus 5% potassium nitrate dentifrice on dentin hypersensitivity, Compend. Cont. Educ. Dent. 37 (2016) 21–25

27- JOINER,A. Tooth color : a review of the literature. Journal of Dentistry,v.32, p.3-12, 2004.

28. GERNHARDT CR. How valid and applicable are current diagnostic criteria and assessment methods for dentin hypersensitivity? An overview Clin Oral Investig 2013; 17(Suppl 1): S31– S40. 5.

29. BAMISE CT, ESAN TA. Mechanisms and treatment approaches of dentine hypersensitivity: a literature review. Oral Health Prev Dent 2011; 9: 353–367. 6. PASHLEY DH. Dentine permeability and its role in the pathobiology of dentine sensitivity. Arch Oral Biol 1994; 39(Suppl): 73S–80S.

30. CORTIANO FM, RACHED RN, MAZUR RF, VIEIRA S, FREIRE A, DE SOUZA EM. Effect of desensitizing agents on the microtensile bond strength of two-step etch-and-rinse adhesives to dentin. Eur J Oral Sci 2016; 124: 309–315

31. CUNHA-CRUZ J, STOUT JR, HEATON LJ, WATAHA JC, NORTHWEST P. Dentin hypersensitivity and oxalates: a systematic review. J Dent Res 2011; 90: 304–310.

32. ANTONIAZZI RP, MACHADO ME, GRELLMANN AP, SANTOS RC, ZANATTA FB. Effectiveness of a desensitizing agent for topical and home use for dentin hypersensitivity: a randomized clinical trial. Am J Dent 2014; 27: 251–257.

33. HAN L, OKIJI T. Dentin tubule occluding ability of dentin desensitizers. Am J Dent 2015; 28: 90–94.

34. CUENIN MF, SCHEIDT MJ, O’NEAL RB, STRONG SL, PASHLEY DH, HORNER JA, VAN DYKE TE. An in vivo study of dentin sensitivity: the relation of dentin sensitivity and the patency of dentin tubules. J Periodontol 1991; 62: 668–673.

35. PASHLEY DH, O’MEARA JA, KEPLER EE, GALLOWAY SE, THOMPSON SM, STEWART FP. Dentin permeability. Effects of desensitizing dentifrices in vitro. J Periodontol 1984; 55: 522–525.

36. GREENHILL JD, PASHLEY DH. The effects of desensitizing agents on the hydraulic conductance of human dentin in vitro. J Dent Res 1981; 60: 686–698.

37. PEREIRA JC, SEGALA AD, GILLAM DG. Effect of desensitizing agents on the hydraulic conductance of human dentin subjected to different surface pre-treatments–an in vitro study. Dent Mater 2005; 21: 129–138.

38. MUZZIN KB, JOHNSON R. Effects of potassium oxalate on dentin hypersensitivity in vivo. J Periodontol 1989; 60: 151–158.

39- DUFOSSÉ,L. MABON,P. BINET,E. Assesment of the coloring strengh of Brevibacterium linens starains: spectrocolorimetry X total carotenoid Extraction quantification. J. Daiary Sci v.84. p. 354-360. American Dairy Science Association. 2001.

40- DAHL,J.E. ; PALLESEN,U.; TOOTH BLEACHING—A CRITICAL REVIEW OF THE BIOLOGICAL ASPECTS. Crit Rev Oral Biol Med.v. 14 n.4 p.292-304, 2003.

41. World Medical Association. (2013). World Medical Association Declaration of Helsinki: ethical principles for medical research involving human subjects. [*Journal of the American Medical Association*](https://pt.wikipedia.org/wiki/Journal_of_the_American_Medical_Association), 310(20), 2191

42.Masalu JR, Åstrøm AN. Applicability of an abbreviated version of the oral impacts on daily performances (OIDP) scale for use among Tanzanian students.  Community Dent Oral Epidemiol. 2003;31(1):7-14.

43. Kothari S, Jum’ah AA, Gray AR, Lyons KM, Yap M, Brunton PA. A randomized clinical trial investigating three vital tooth bleaching protocols and associated efficacy, effectiveness and participants’ satisfaction. J Dent. 2020;95(1):103322.

**7** – CRONOGRAMA DE ATIVIDADES

| ATIVIDADES | ANO: 2020 / 2021  MESES | | | | | | | | | | | | | | | |  |
| --- | --- | --- | --- | --- | --- | --- | --- | --- | --- | --- | --- | --- | --- | --- | --- | --- | --- |
|  | A | S | O | N | D | J | F | M | A | M | J | J | A | S | O | N | D |
| Levantamento bibliográfico | X | X | X | X | X | X | X | X | X | X | X | X | X | X | X | X | X |
| Submissão ao comitê de bioética |  |  |  | X | X |  |  |  |  |  |  |  |  |  |  |  |  |
| Triagem dos voluntários | X | X | X |  |  |  |  |  |  |  |  |  |  |  |  |  |  |
| Realização do ensaio clínico | X | X | X | X | X | X | X | X | X | X | X | X | X | X |  |  |  |
| Tabulação dos resultados e análise estatística |  |  |  |  |  |  |  |  |  |  |  |  |  |  | X | X | X |
| Entrega do relatório final do projeto de pesquisa |  |  |  |  |  |  |  |  |  |  |  |  |  |  |  |  | X |
