# Supplementary material for: Effect of 1.5% potassium oxalate on sensitivity control, color change, and quality of life after at-home tooth whitening: A randomized, placebo-controlled clinical trial
Source: PLoS One. 2022 Nov 17;17(11):e0277346. doi: 10.1371/journal.pone.0277346 (PMC9671445; doi:10.1371/journal.pone.0277346)
Supplement: S2 Protocol — (DOC) [file pone.0277346.s003.doc]

**RESEARCH PROJECT**

**1 - PROJECT IDENTIFICATION**

PROJECT TITLE: Effect of 1.5% potassium oxalate in the control of tooth sensitivity and color change after at-home bleaching: a randomized, placebo-controlled clinical study.

GREAT AREA OF KNOWLEDGE: (Health Sciences)

AREA OF KNOWLEDGE: Dentistry (4.02.00.00.0)

SUBAREA: Dentistry (4.02.04.000)

INSTITUTION: Federal University of Pará

CENTER / DEPARTMENT: Health Sciences Center / Faculty of Dentistry

EXECUTIVE UNIT: Faculty of Dentistry

ADDRESS: Av. Augusto Corrêa, nº 01 – José da Silveira Netto University City

| COUNTY  Bethlehem | Zip code  66640480 | U.F.  PA | TEL/FAX  91-32017494 | E-MAIL  [cecymsilva@gmail.com](mailto:cecymsilva@gmail.com) |
| --- | --- | --- | --- | --- |

PROJECT'S COORDENADOR: CECY MARTINS SILVA

DEPARTMENT: SCHOOL OF DENTISTRY

OTHER PARTICIPATING INSTITUTIONS

**2 – PROJECT TEAM**

| **Registration** | **Full name** | **Type*** | **Maximum Degree** | **Department** | **Role in the project**** | **Workload in the project** |
| --- | --- | --- | --- | --- | --- | --- |
| 0327584 | Cecy Martins Silva | PE | Doctor | Dental School | CD | 5h |
| 1259040 | Eliane Bemerguy Alves | PE | Doctor | Dental School | CL | 5h |
|  | Antonia Patricia Oliveira Barros |  | Scientific Initiation Student | Dental School | CL | - |
|  | Danielle da Silva Pompeu |  | Scientific Initiation Student | Dental School | CL | - |
|  | Elma Vieira Takeuchi |  | Master's Degree student | Dental School | CL | - |
|  | Cristiane de Melo Alencar |  | PhD student | Dental School | CL | - |

* AT: Admnistrative technician ** CD: Coordinator

VP: Visiting Professor CL: Collaborator

PE: Permanent Professor (located in the center where the project belongs) CS: Consultant

PP: Participating Professor (located at another center) EPP: External Participating Professor

ET: External Administrative Technician

SP: Scholarship Professor at a Development Agency (CAPES, CNPQ, DAAD, etc..)

**RESEARCH PROJECT**

**3 – INTRODUCTION**

Tooth whitening is considered a conservative treatment to remove pigments that promote unwanted darkening of the teeth, ^1,2,3^ and it is recommended to treat patients who seek improvements in dental appearance, as it presents a minimally invasive approach when compared to other treatments. invasive aesthetics. ^4^ Carbamide peroxide (PC) is one of the active components used in the whitening gel, which is available in different concentrations.^1,5^ The oxidizing agent present in PC is capable of diffusing through dental enamel and dissociating into unstable free radicals. (perhydroxyl, hydroxyl and oxygen) which react with the macromolecules of organic pigments, triggering the breakage of the pigments' carbon double bonds, resulting in smaller molecules that provide a change in the absorption spectrum, leaving the tooth lighter. ^5^

A very common unwanted effect during home bleaching treatment is tooth sensitivity.^6,7,8,9^ This sensitivity is reported to occur, generally, in the first weeks of treatment.^10,11^ This sensitivity is related to the amount of free radicals. free of carbamide peroxide that reach the pulp through the dentinal tubules.^12,2,13,14^ To reduce the pain caused by the bleaching treatment, some methods can be used, such as the use of gels with lower concentration and/or reduction of time. , frequency of application of the whitening gel,^12,2^ administration of analgesics/anti-inflammatory drugs^15^ and the use of desensitizers.^5,9,16,3^

Many agents with different mechanisms of action have been described and evaluated in the literature related to the management of HD. Dentin desensitizers can be classified, according to their action, as neural ones (e.g. potassium nitrate and low power lasers), which act on nerve cell stimulation, more specifically the Na + / K + pump in the membrane cell membrane, interfering with cell membrane polarity, increasing membrane action potential amplitude, and blocking the transmission of painful stimuli, ^17^ dentinal tubule blockers, for example, oxalates, glutaraldehyde and high-power lasers, in which they generate precipitates capable of occluding open dentinal tubules below the surface, interfering with the hydrodynamics of the dentinal fluid, thus preventing dentin sensitivity,^18^ and agents with both actions (e.g. potassium oxalate), in which oxalate has the action of forming insoluble precipitates in the dentinal tubules blocking the movement of dental fluid, and potassium that acts to reduce the transmission of nerve impulses .^19^

Among desensitizers, potassium oxalate has been widely used in clinical practice, with satisfactory results, without side effects.^20,21,22^ The mechanism of action of this agent is based on the obliteration of exposed dentin tubules, due to precipitation of crystals. oxalate ^23^ and depolarization of nerve endings.^17^ In a systematic review of the literature, in which the effectiveness of different desensitizing agents in reducing dentin sensitivity was evaluated, it was observed, based on the results of the authors, ^24^ that oxalate potassium had a significant effect on pain reduction. To date, there are no reports in the literature of clinical studies that evaluated the effect of 1.5% potassium oxalate in the control of sensitivity after home bleaching with 22% carbamide peroxide, justifying the objectives of this scientific investigation.

Tooth color is considered a phenomenon of great complexity influenced by factors such as ambient lighting conditions, translucency, opacity, light scattering, brightness and also by the structures and morphophysiological processes of vision that vary in each individual.^25^ Color perception it is subjective and needs more quantitative support to be evaluated. The spectrophotometer is an equipment capable of measuring the wavelength of a given radiation from the reflectance or transmittance of an object.26 In this study, we will use the EasyshadeAdvanced spectrophotometer (Vita-Zahnfabrik, Germany), using the CIE L*a system *b*, to assess color change between groups bleached with 22% carbamide peroxide. It is worth mentioning that the CIEL*a*b* color system is capable of converting the human eye's response to colors, so that it is electronically measured. ^26^

**4 – JUSTIFICATION**

Oxalates, in particular, have a long history in the treatment of HD ^27,28,29^ and are widely recognized as capable of reducing postoperative sensitivity.^30^ Treatment with oxalate-based products is capable of decreasing dentin permeability. ,^31,32,33^ form precipitates in the dentin tubules,^34^ block fluid flow ^35,36^ and reduce HD-related pain.

Oxalate precipitates are also relatively insoluble in acid, making them resistant to dissolution in the oral environment.^37^ The combination of potassium and oxalates was introduced for DH in the late 1970s^31^ and produced two main effects: an initial effect, from potassium ions, characterized by neutral depolarization; and late effect, resulting from obstruction of the tubules by the formation of calcium oxalate crystals.^38^ The latter is based on the exchange of ions between the metal ions present in the oxalate (potassium) and those (Ca2+) in the dentinal fluid. The exchange of Ca2+ for K+ leads to the formation of a 'slightly soluble' calcium oxalate crystalline layer, which is deposited on the dentin surface.

The color system – CIE L*a*b*, which will be used in this study, is based on the theory of color perception of the three receptors in the eyes (three types of retinal cones) separated by the primary colors – red, green , blue.^26^ Through this system the reflectance spectrum is filtered so that the spectrum of the red, green and blue hue is extracted as three coordinates, and processed to give the answer L*a*b*.^39^ The letters of the acronym CIE L*a*b* relates to the three directions in the color sphere: L* stands for color luminosity, the chromatic coordinates a*(represent red–green) and b* (represent yellow–blue), are perpendicular to L* and they leave in four orthogonal directions (a+): characterizing the reddish color; (a-): characterizing the color green; (b+): characterizing a tendency to yellow; and (b-): with a color tendency towards blue hue.^26,27^

In addition to the a* and b* attributes, their combination also determines Chroma (C*) and H* (Hue) parameters.^27^ Chroma is the color saturation, it measures the distance from the L* axis in that system and represents the change of a opaque, duller state to a saturated, vivid color. Hue (H *) is an angle measure that determines the predominant wavelength that makes up that color (predominant hue). ^39^ Color can be determined by a combination of intrinsic and extrinsic staining effects, the first related to light scattering and the absorption properties of enamel and dentin, determined by their morphophysiological conditions, and the other associated with the absorption of pigments on the surface of the tooth. tooth enamel. ^40^

This randomized, placebo-controlled clinical study will therefore positively contribute to the elucidation of the effectiveness of 1.5% potassium oxalate in controlling pain sensitivity after home bleaching treatment, as well as in the perception of color change between the groups. bleached with 22% carbamide peroxide.

**RESEARCH PROJECT**

**5 – OBJECTIVE**

This randomized, placebo-controlled clinical trial aims to evaluate the effect of 1.5% potassium oxalate in controlling pain sensitivity and color change after at-home tooth whitening. The null hypotheses tested in the present study will be: H01 - There will be no difference in tooth sensitivity between the groups bleached with 22% carbamide peroxide associated or not with 1.5% potassium oxalate, thirty days after the end of treatments.

H02- There will be no difference in color change between the groups bleached with 22% carbamide peroxide associated or not with 15% potassium oxalate, thirty days after the end of treatments.

H03- Use of 1.5% potassium oxalate after bleaching treatment will not influence Health-Related Quality of Life (HRQoL).

H04- Use of potassium oxalate after bleaching treatment will not influence patient satisfaction.

**6 – METHODOLOGY**

## **6.1. Ethical aspects**

## This research project will follow the recommendations of the “consort” (consolidated standards of reporting trials). The research volunteers will be duly clarified and informed about the risks, methods and objectives of this project, being necessary the signature of the free and informed consent term – tcle (Annex I), in accordance with the Helsinki Declaration.^41^

## All information to be collected will have an exclusively scientific purpose and the identity of the volunteers will be preserved. Participation in the study may be canceled and the consent form withdrawn at any time during the research, ensuring the confidentiality of the volunteer's confidentiality even in case of abandonment of treatment.

## **6.2. Sample selection**

Fifty volunteers aged between 18 and 29 will be selected, who must follow the inclusion and exclusion criteria described in table 1. The clinical evaluation of the volunteers will be carried out through anamnesis, intraoral clinical examination and color registration. All participants will undergo prophylaxis performed with a rubber cup (Microdon, SP, Brazil) and pumice stone (Biodinamic, PR, Brazil) seven days before the start of the study and will receive oral hygiene kits, to be used throughout the treatment, for the standardization of a toothpaste that does not have a desensitizing action and that does not contain fluor, in order to mitigate possible interferences in the evaluation of this study. The kit will consist of a toothbrush (Oral B, Cerdas Indicator, São Paulo, SP, Brazil) and a toothpaste (My First Colgate®, Colgate-Palmolive Company, SP, Brazil), under instructions for use three times a day.

Table 1: Inclusion and exclusion criteria.

| ***INCLUSION CRITERIA*** | ***EXCLUSION CRITERIA*** |
| --- | --- |
| - Shade greater than A2 in upper incisors and canines, according to the Vita Classical shade guide; - Absence of active caries lesions; - Patients who have never undergone bleaching therapy - Present good oral hygiene; - Not presenting hypersensitivity to tactile and evaporative stimuli through the Visual Analog Pain Scale; - Not being a smoker; - Not being pregnant; | - Patients undergoing fixed orthodontic treatment; - Non-vital teeth with darkening; - Presence of cracks or fractures; - Patients allergic to the product; - Extensive restorations in molars; - Presence of gastroesophageal dysfunctions; - Presence of dentinal exposure in anterior and/or posterior teeth; - Patients over 30 years of age. |

## **6.3. Study design**

## This clinical trial will be randomized, placebo-controlled and blinded. To determine the groups, a randomization process will be carried out to determine the treatment to be applied. Patients will be randomized and randomly divided into two groups: G1- Placebo; G2 – Potassium Oxalate; as described in figure 1.

Evaluated Patients (N=X)

Excluded Patients Y=(X-50)

**Recruitment**

Randomization by lottery (N=50)

Whitening treatment

Polanight 22% Carbamide Peroxide (SDI)

**Follow-up**

**G1 (n=25)**

Placebo

(fluoride-free toothpaste)

**G2 (n=25)**

Experimental Group

(1.5% Potassium Oxalate)

Pain assessment by daily questionnaire

(n=50)

**Analysis**

**Figure 1: Study design**

All groups will undergo home bleaching treatment with 22% Polanight carbamide peroxide (SDI). Only the G2 group will receive desensitizing treatment with 1.5% potassium oxalate gel (Table 2). Volunteers will be instructed on the use of whitening treatment and treatment with placebo or desensitizing.

**Table 2.** Division of groups, bleaching treatment, desensitizing treatments.

| ***GROUPS (N=50)*** | ***BLEACHING TREATMENT*** | ***DESENSITIZING TREATMENT*** |
| --- | --- | --- |
| **G1** |  | ---- |
| **G2** | Pola Night CP 22%  (SDI) | 1.5% Potassium Oxalate (Painless, BM4,SC, Brazil) |

**6.4. Randomization**

The randomization process will be carried out by numerical drawing by the main researcher. A number will be assigned to each experimental group (1 for G1, 2 for G2) and a lottery will be performed for each volunteer. Patients will be numbered according to the enrollment sequence.

**6.5. Blinding**

In this double-blind study, the participant will be unaware of the desensitizing treatment he will receive, as both the desensitizing gel and the placebo gel will be placed in the same container to make their identification impossible. The texture, color and odor of the placebo will be similar to 1.5% potassium oxalate (Painless, BM4, SC, Brazil). The pain sensitivity evaluator will also be unaware of the group to which the participant will belong, because he will not participate in the randomization process. The research will have a single operator, who will perform the experimental part.

**6.6 Intervention**

Each volunteer in the group will receive a kit containing a tube with Polanight bleaching agent (SDI, SP, Brazil), two individual trays of ethylene/vinyl acetate copolymer (upper and lower), an unmarked tube with the desensitizer (potassium oxalate at 1.5%), a toothbrush and a fluoride-free toothpaste (My First Colgate®, Colgate-Palmolive Company, SP, Brazil).

*-Dental whitening*

The volunteers will be molded with alginate (Jeltrate Plus - Dentsply) with the aid of a tray (Tecnodent). Then, the models will be made using special plaster (Durone-Dentsply). The trays will be made from the models obtained using an ethylene/vinyl acetate copolymer (FGM) plate and a vacuum plasticizer (Plastivac P7/Bio Art). It will be recommended to apply a drop of the whitening agent Polanight (SDI, SP, Brazil) on each corresponding tooth in the tray that should be used for 45 minutes a day for a period of twenty one days.

- *Placebo*

After the bleaching treatment, the trays will be washed with running water, and the volunteers in group 1 will apply a small amount of the placebo gel, without active ingredient, with a color, texture and odor similar to 1.5% oxalate gel (Painless, BM4, SC, Brazil) in the spaces related to the buccal portions of the teeth of the trays, which should be used for 10 minutes. After removing the tray, the patient should brush the teeth with the fluoride-free toothpaste (My First Colgate®, Colgate-Palmolive Company, SP, Brazil).

*- Treatment with 1.5% Potassium Oxalate*

After the bleaching treatment, the trays will be washed with running water, and the volunteers in group 1 will apply a small amount of 1.5% oxalate gel (Painless, BM4, SC, Brazil) in the spaces related to the buccal portions of the teeth of the teeth trays, which should be used for 10 minutes. After removing the tray, the patient should brush their teeth with fluoride-free toothpaste (My First Colgate®) and clean the tray in order to remove all the gel inside the tray.

**6.9. Color Assessment**

Color evaluation will be performed on the upper incisors and canines of each volunteer with an EasyshadeAdvanced spectrophotometer (Vita-Zahnfabrik, Germany), using the CIE L*a*b* system, where the values ​​of color change (ΔE) were obtained for each group of teeth using the formula: ΔE = {(ΔL)^2^ + (Δa)^2^ + (Δb)^2^}^1/2^ , where: ΔL* = L*- L*_0_; Δa* = a*-a*_0_; and Δb* = b*- b*_0_. The color evaluation will be performed at three times: before the whitening treatment serving as a baseline, 21 days and 1 month after the last application of the whitening gel.

## **6.10. Sensitivity assessment**

## Postoperative sensitivity will be evaluated through a daily questionnaire that will be provided to volunteers from the first at-home bleaching treatment session, which must be completed during the 21 days of treatment, according to their personal perception and pain threshold, on the level of sensitivity or discomfort caused by the bleaching treatment, on a scale from 0 (no pain) to 10 (severe pain), according to Visual Analogue Scale.

## **6.11. Assessment of Oral Impact on Daily Performance (OIDP)**

## The OIDP will be used to measure the oral impact caused by bleaching treatment on individuals' ability to perform daily activities. It includes nine performances to be evaluated, based on the model by Masalu (2003),^42^ which analyzes physical, psychological and social activities, including eating, speaking and pronouncing correctly; teeth cleaning; sleep and relax; smiling, laughing and showing teeth without feeling ashamed and maintaining the emotional state without getting angry. For each reported impact, the patient will record the main symptom (1- tooth sensitivity, 2- tooth color, or 0- other reason). This questionnaire will be provided before and after the whitening treatment.

## **6.12. Assessment of the degree of patient satisfaction with the bleaching treatment**

## At the end of treatment, patients will be asked to express themselves using a 7-point scale, based on the modified model by Kothari et al. (2020),^43^ the degree of satisfaction with the treatment. The scale will be graded from 1 (not at all satisfied) to 7 (very satisfied). Participants will also use the scale to express whether they would recommend the whitening protocol to their family and friends, choosing from 1 (would not recommend) to 7 (would recommend). Volunteers will also answer a questionnaire with five questions about their satisfaction with the whitening protocol used and the results achieved with the treatment. For each question, the volunteers will indicate one of the following scores: 1- I totally agree; 2 - I partially agree; 3 - no opinion; 4 - I somewhat disagree and 5 - I totally disagree.

## **6.13. Statistical analysis**

## The sensitivity values ​​reported by the volunteers will be tabulated in an Excel spreadsheet (Microsoft Windows 2010) and analyzed using the BioEstat.® program. If this study generates non-parametric data, the Friedman test will be performed for intragroup analysis and the Wilcoxon test for intergroup analysis. For all analyses, significance levels of 5% will be considered.

**RESEARCH PROJECT**

**7 – GOALS**

**-** The bibliographic survey will take place from August 2020 to December 2021;

- After approval by the Bioethics Committee, a randomized clinical trial will begin: the recruitment, randomization and allocation of volunteers, treatment, sensitivity assessment and color change will take place from August 2020 to September 2021.

- Tabulation and statistical analysis of the results will be carried out in the period between October and December 2021;

- The preparation and delivery of the final report will take place until December 2021

**8 - BIBLIOGRAFIA**

1. Abouassi T, Wolkewitz M, Hahn P. Effect of carbamide peroxide and hydrogen peroxide on enamel surface: an in vitro study. Clin Oral Invest. 2011; 15:673–680.

2. Basting RT, Amaral FLB, França FMG, Flório FM. Clinical comparative study of the effectiveness of and tooth sensitivity to 10% and 20% carbide peroxide home-use and 35% and 38% hydrogen peroxide in-office bleaching materials containing desensitizing agents. Oper Dent. 2012; 37(5):464- 473.

3. Tay LY, Kose C, Loguercio AD, Reis A. Assessing the effect of a desensitizing agent used before in-office tooth bleaching. JADA. 2009; 140(10): 1245-1251.

4 Alani A, Kelleher M, Hemmings K, Saunders M, Hunter M,Barclay S, Ashley M, Djemal S, Bishop K, Darbar U (2015)Balancing the risks and benefits associated with cosmetic dentistry–a joint statement by UK specialist dental societies. Br Dent J218(9):543–548. https://doi.org/10.1038/sj.bdj.2015.345.

5Alqahtani MQ. Tooth-bleaching procedures and their controversial effects: A literature review. Saudi Dent J. 2014; 26:33-46.

6. Bonaf E, Bacovis LB, Iensen S, Loguercio AD, Reis A, Kossatz S. Tooth sensitivity and efficacy of in-office bleaching in restored teeth. J Dent. 2013; 41:363-369 .

7. Haywood VB, Leech T, Heymann HO, Crumpler D, Bruggers K. Nightguard vital bleaching: effects on enamel surface texture and diffusion. Oper Dent. 1990; 21(10): 801-804.

8. Kossatz S, Dalanhol AP, Cunha T, Loguercio A, Reis A. Effect of light activation on tooth sensitivity after in-office bleaching. Oper Dent. 2011; 36(3): 251-257.

9. Reis A, Dalanhol AP, Cunha TS, Kossatz S, Loguercio AD. Assessment of tooth sensitivity using a desensitizer before light-activated bleaching. Oper Dent. 2011; 36(1): 12-17.

10. Browning WD, Cho SD, Deschepper EJ. Effect of a nano- hydroxyapatite paste on bleaching related tooth sensitivity. J Esthet Restor Dent. 2012; 24(4):268-276.

11. Po LH & Wilson N. Effects of differents desensitizing agents on bleaching treatments. EJGD. 2014; 3(2): 93-99.

12. Armênio RV, Fitarelli F, Armênio MF, Demarco FF, Reis A, Loguercio AD. The effect of fluoride gel use on bleaching sensitivity: a doubleblind randomized controlled clinical trial. JADA. 2008; 139(5):592-597.

13. Camargo SEA, Valera MC, Camargo CHR, Mancini MNG, Menezes MM. Penetration of 38% hydrogen peroxide into the pulp chamber in bovine and human teeth submitted to office bleach technique. JOE. 2007; 33(9):1074-1077.

14. Costa CAS, Riehl H, Kina JF, Sacono NT, Hebling J. Human pulp response to in-office tooth bleaching. Oral Surg Oral Med Oral Pathol Oral Radiol Endod. 2010; 109:59-64.

15. Charakorn P, Cabanilla LL, Wagner WC, Foong WC, Shaheen J, Pregitzer R, Schneider D. The effect of preoperative ibuprofen on tooth sensitivity caused by in-office bleaching. Oper Dent. 2009; 34:131–135.

16. Cerqueira RR, Hofstaetter FL, Rezende M, Martins GC, Loguercio AD, Reis A, Kossatz S. Efeito do uso de agente dessensibilizante na efetividade do clareamento e na sensibilidade dental. Rev Assoc Paul Cir Dent. 2013; 67(1):64-67.

17- Wakabayashi H, Hamba M, Matsumoto K, Tachibana H (1993) Effect of irradiation by semiconductor laser on responses evoked in trigeminal caudal neurons by tooth pulp stimulation. Lasers Surg Med 13:605–610

18. A. Davari, E. Ataei, H. Assarzadeh, Dentin hypersensitivity: etiology, diagnosis and treatment; a literature review, J. Dent. 14 (3) (2013) 136–145.

19. E. Oncu, S. Karabekiroglu, N. Unlu, Effects of different desensitizers and lasers on dentine tubules: an in-vitro analysis, Microsc. Res. Tech. 80 (7) (2017) 737–744

20. D.G. Gillam, H.S. Seo, H.N. Newman, J.S. Bulman, Comparison of dentine hypersensitivity in selected occidental and oriental populations, J. Oral Rehabil. 28 (1) (2001) 20–25.

21. J. Pereira, A. Martineli, S. Santiago, Treating hypersensitive dentin with three different potassium oxalate-based gel formulations: a clinical study, J. Appl. Oral Sci. 9 (2001) 123–130.

22. S. Sauro, M.G. Gandolfi, C. Prati, R. Mongiorgi, Oxalate-containing phytocomplexes as dentine desensitisers: an in vitro study, Arch. Oral Biol. 51 (8) (2006) 655–664.

23. E.M. Varoni, T. Zuccheri, A. Carletta, B. Palazzo, A. Cochis, M. Colonna, L. Rimondini, In vitro efficacy of a novel potassium oxalate hydrogel for dentin hypersensitivity, Eur. J. Oral Sci. 125 (2) (2017) 151–159.

24- Ayad F, Ayad N, Yun Po Zhang YP, De Vizio W, Cummis D, Mateo LR. Comparing the efficacy in reducing dentin hypersensitivity of a new toothpaste containing 8.0% Arginine Calcium Carbonate, and 1450 ppm fluoride to a commercial sensitive toothpaste containing 2% potassium ion: an eight-week clinical study on Canadian adults. J Clin Dent. 2009;20(1):10-6.

25 -JOINER,A. Tooth color : a review of the literature. Journal of Dentistry,v.32, p.3-12, 2004.

26. P. Amini, M. Miner, P.A. Sagel, R.W. Gerlach, Effects of 1.5% oxalate stripes versus 5% potassium nitrate dentifrice on dentin hypersensitivity, Compend. Cont. Educ. Dent. 37 (2016) 21–25

27- JOINER,A. Tooth color : a review of the literature. Journal of Dentistry,v.32, p.3-12, 2004.

28. GERNHARDT CR. How valid and applicable are current diagnostic criteria and assessment methods for dentin hypersensitivity? An overview Clin Oral Investig 2013; 17(Suppl 1): S31– S40. 5.

29. BAMISE CT, ESAN TA. Mechanisms and treatment approaches of dentine hypersensitivity: a literature review. Oral Health Prev Dent 2011; 9: 353–367. 6. PASHLEY DH. Dentine permeability and its role in the pathobiology of dentine sensitivity. Arch Oral Biol 1994; 39(Suppl): 73S–80S.

30. CORTIANO FM, RACHED RN, MAZUR RF, VIEIRA S, FREIRE A, DE SOUZA EM. Effect of desensitizing agents on the microtensile bond strength of two-step etch-and-rinse adhesives to dentin. Eur J Oral Sci 2016; 124: 309–315

31. CUNHA-CRUZ J, STOUT JR, HEATON LJ, WATAHA JC, NORTHWEST P. Dentin hypersensitivity and oxalates: a systematic review. J Dent Res 2011; 90: 304–310.

32. ANTONIAZZI RP, MACHADO ME, GRELLMANN AP, SANTOS RC, ZANATTA FB. Effectiveness of a desensitizing agent for topical and home use for dentin hypersensitivity: a randomized clinical trial. Am J Dent 2014; 27: 251–257.

33. HAN L, OKIJI T. Dentin tubule occluding ability of dentin desensitizers. Am J Dent 2015; 28: 90–94.

34. CUENIN MF, SCHEIDT MJ, O’NEAL RB, STRONG SL, PASHLEY DH, HORNER JA, VAN DYKE TE. An in vivo study of dentin sensitivity: the relation of dentin sensitivity and the patency of dentin tubules. J Periodontol 1991; 62: 668–673.

35. PASHLEY DH, O’MEARA JA, KEPLER EE, GALLOWAY SE, THOMPSON SM, STEWART FP. Dentin permeability. Effects of desensitizing dentifrices in vitro. J Periodontol 1984; 55: 522–525.

36. GREENHILL JD, PASHLEY DH. The effects of desensitizing agents on the hydraulic conductance of human dentin in vitro. J Dent Res 1981; 60: 686–698.

37. PEREIRA JC, SEGALA AD, GILLAM DG. Effect of desensitizing agents on the hydraulic conductance of human dentin subjected to different surface pre-treatments–an in vitro study. Dent Mater 2005; 21: 129–138.

38. MUZZIN KB, JOHNSON R. Effects of potassium oxalate on dentin hypersensitivity in vivo. J Periodontol 1989; 60: 151–158.

39- DUFOSSÉ,L. MABON,P. BINET,E. Assesment of the coloring strengh of Brevibacterium linens starains: spectrocolorimetry X total carotenoid Extraction quantification. J. Daiary Sci v.84. p. 354-360. American Dairy Science Association. 2001.

40- DAHL,J.E. ; PALLESEN,U.; TOOTH BLEACHING—A CRITICAL REVIEW OF THE BIOLOGICAL ASPECTS. Crit Rev Oral Biol Med.v. 14 n.4 p.292-304, 2003.

41. World Medical Association. (2013). World Medical Association Declaration of Helsinki: ethical principles for medical research involving human subjects. [*Journal of the American Medical Association*](https://pt.wikipedia.org/wiki/Journal_of_the_American_Medical_Association), 310(20), 2191

42.Masalu JR, Åstrøm AN. Applicability of an abbreviated version of the oral impacts on daily performances (OIDP) scale for use among Tanzanian students.  Community Dent Oral Epidemiol. 2003;31(1):7-14.

43. Kothari S, Jum’ah AA, Gray AR, Lyons KM, Yap M, Brunton PA. A randomized clinical trial investigating three vital tooth bleaching protocols and associated efficacy, effectiveness and participants’ satisfaction. J Dent. 2020;95(1):103322.

**7 – SCHEDULE OF ACTIVITIES**

| ACTIVITIES | ANO: 2020 / 2021  MONTHS | | | | | | | | | | | | | | | |  |
| --- | --- | --- | --- | --- | --- | --- | --- | --- | --- | --- | --- | --- | --- | --- | --- | --- | --- |
|  | A | S | O | N | D | J | F | M | A | M | J | J | A | S | O | N | D |
| Bibliographic survey | X | X | X | X | X | X | X | X | X | X | X | X | X | X | X | X | X |
| Submission to the bioethics committee |  |  |  | X | X |  |  |  |  |  |  |  |  |  |  |  |  |
| Volunteer screening | X | X | X |  |  |  |  |  |  |  |  |  |  |  |  |  |  |
| Conducting the clinical trial | X | X | X | X | X | X | X | X | X | X | X | X | X | X |  |  |  |
| Tabulation of results and statistical analysis |  |  |  |  |  |  |  |  |  |  |  |  |  |  | X | X | X |
| Delivery of the final report of the research project |  |  |  |  |  |  |  |  |  |  |  |  |  |  |  |  | X |
